# Cocaine-Induced Bilateral Basal Ganglia Infarction in a Patient With ST-Elevation Myocardial Infarction: A Case Report

**DOI:** 10.7759/cureus.81099

**Published:** 2025-03-24

**Authors:** Marisol Trejos, Solomon Nittala, Kester Nedd

**Affiliations:** 1 Neurology, Larkin Community Hospital Palm Springs Campus, Hialeah, USA

**Keywords:** acute cocaine intoxication, basal ganglia infarction, bilateral basal ganglia hyperintensities, cocaine-induced myocardial infarction, cocaine-induced thrombosis, symmetrical basal ganglia (deep grey matter nuclei) lesions

## Abstract

Cocaine is an alkaloid-based extract made into cocaine hydrochloride, a substance found to influence the central nervous system to a higher degree when smoked and linked to numerous neurological ailments such as stroke, hemorrhage, seizures, and other cognitive impairments. We present the unique case of a 60-year-old male with unknown past medical history presenting initially with an acute ST-elevation myocardial infarction (STEMI) and a concurrent cardiocerebral infarction (more specifically in the bilateral globus pallidus stroke), with toxicology positive for cocaine intoxication.

Lesions in the bilateral basal ganglia are usually correlated to carbon monoxide poisoning, cardiorespiratory arrest, hypovolemia, trauma, heroin usage, and methanol intoxication. However, in extremely rare instances, cocaine usage can lead to cerebral vasospasm causing infarction. Vascular thrombosis can be caused by platelet aggregation potentiated by cocaine. This unique presentation of bilateral basal ganglia with the comorbidity of a STEMI in association with cocaine intoxication encourages further research toward the usage of innovative imaging techniques such as positron emission tomography and single-photon emission computed tomography to facilitate viewing lesions associated with cerebral blood volume flow in cases where substance intoxication play a preeminent factor.

## Introduction

Cocaine is an alkaloid-based extract made into a hydrochloride molecule. The substance can be ingested intranasally, within the mucous membranes of the gum line, or even smoked in crack-cocaine forms. Smoking this form of cocaine has been found to influence the central nervous system to a higher degree than intranasal ingestion. Cocaine has been found to cause stroke, hemorrhage, seizures, and other cognitive impairments [1]. It is understood that cocaine can be the cause of many pathological manifestations; however, cerebral infarction has been a complication known in cocaine use but can be a rare finding [1].

The mechanisms by which cocaine may cause vascular injury both in the brain and the heart are remarkably similar [2]. However, a bilateral and symmetrical ischemic presentation is not a very common finding in patients presenting with positive urine toxicology for cocaine. The case presented demonstrates how the use of certain medications (i.e., phenobarbital) as life-saving measures can further advance injury via vasospasm in patients presenting with ischemic cerebrovascular injuries and myocardial infarction in the setting of cocaine use [1-3]. In this case, the patient was administered phenobarbital which may have further induced or exacerbated vasospasm due to interactions with the cocaine already in his system.

It is also important to highlight the efficacy of newer imaging modalities in the identification of structural lesions, such as single-photon emission computed tomography (SPECT) and positron emission tomography (PET), and the impact it may have on diagnosis in individuals presenting with cerebrovascular injuries in the setting of cocaine use [2,3].

## Case presentation

A 60-year-old male with an unknown past medical history was transferred in from the Lower Keys Medical Center to Kendal Regionals Medical Center with the chief complaint of altered sensorium after a cardiac arrest. Initially, at the Keys Medical Center, the patient was found to be breathing and clinically stable. However, he went into respiratory distress and was intubated for approximately five minutes. He was noted to have had an acute ST-elevation myocardial infarction (STEMI). Due to the patient’s proximity to appropriate care, he was given tenecteplase and transferred to Kendall Regional Medical Center, where he was admitted to the intensive care unit (ICU). Personal medical history could not be obtained due to the patient’s current mental status, with a physical examination being remarkable for an obtunded mental state, pupils equal and reactive, brain stem reflexes intact, and not withdrawing any extremity to noxious stimuli. The patient had no family contact information listed in his belongings as well.

After further investigation with imaging, the patient was found to have a bilateral globus pallidus infarct with initial suspicion of carbon monoxide intoxication or methanol intoxication. However, toxicology, as seen in Table 1, confirmed cocaine intoxication. The patient was also treated for his comorbidities of human immunodeficiency virus, hepatitis C, and acute kidney injury. Less than a month after being in the ICU, the patient succumbed to multiorgan failure and was pronounced dead.

**Table 1:** Urine drug screen. Urine toxicology positive for cocaine intoxication.

Test	Result	Reference
Urine opiates screen	Negative	Negative
Urine barbiturates screen	Negative	Negative
Urine phencyclidine screen	Negative	Negative
Urine amphetamines screen	Negative	Negative
Phenobarbital	Positive	Negative
Urine benzodiazepines screen	Negative	Negative
Urine cocaine screen	Positive	Negative
Urine cannabinoids screen	Negative	Negative

**Figure 1:**
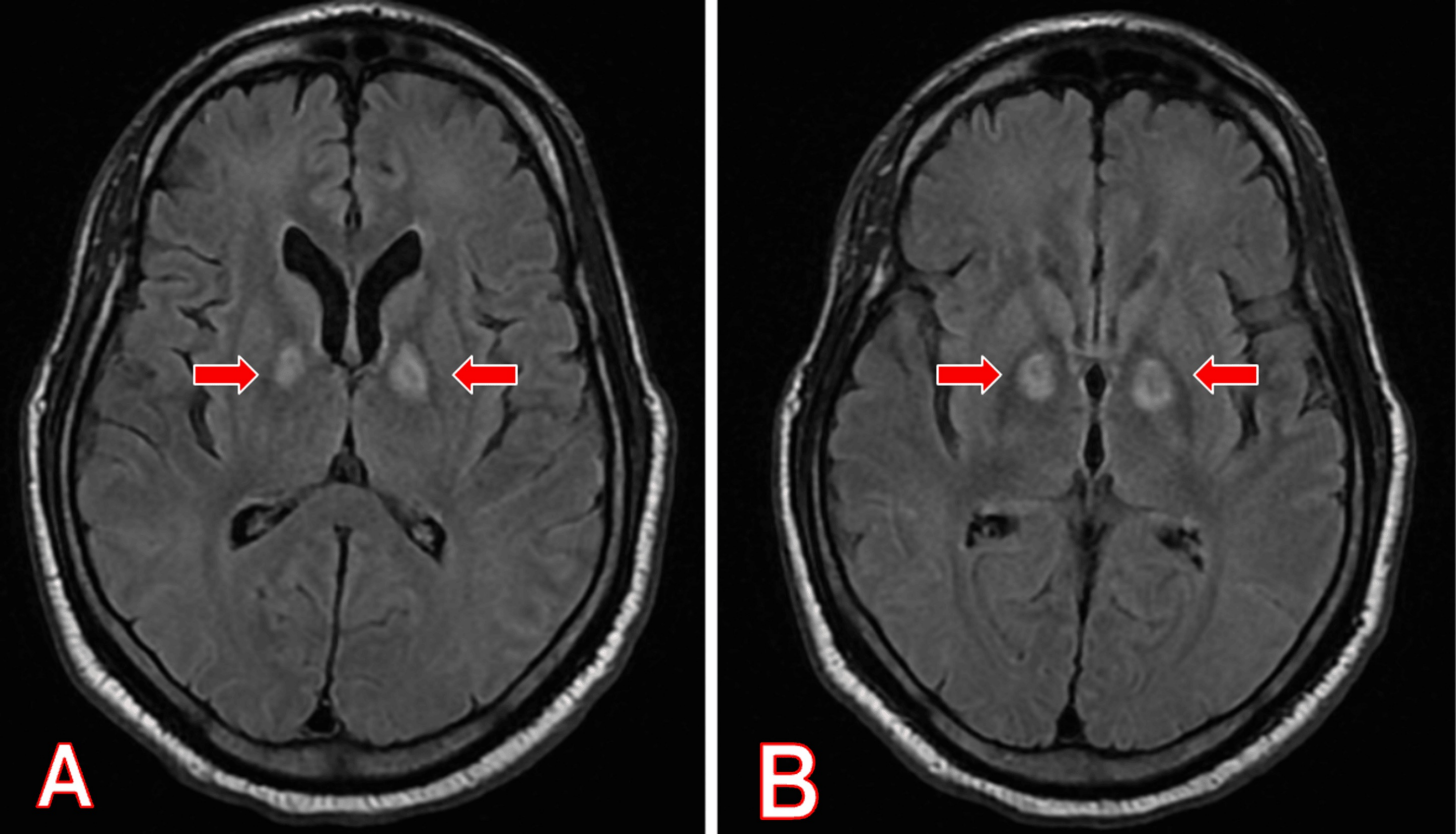
MRI brain without contrast, T2 FLAIR sequence, axial view, demonstrating bilateral basal ganglia infarction. Red arrows show bilateral basal ganglia infarctions on MRI brain without contrast, T2 FLAIR sequence on axial view. (A) First series. (B) Second series. FLAIR: fluid-attenuated inversion recovery

Magnetic resonance imaging of the brain revealed two areas of increased T2/fluid-attenuated inversion recovery (FLAIR) signal within the medial aspect of both basal ganglia, as seen in Figure 1. This imaging led us to suspect bilateral globus pallidus subacute cerebral infarction from cocaine abuse due to hypoperfusion. Johnson et al. gave evidence of the hypoperfusion present in the hippocampal, globus pallidus, and basal ganglia areas through the use of SPECT while intravenous cocaine was administered [2,3].

## Discussion

Currently, 2.4% of the population reports cocaine use in the previous month. Yet, bilateral basal ganglia lesions due to cocaine usage are extremely rare [2,3]. More often than not, they are due to carbon monoxide poisoning, cardiorespiratory arrest, hypovolemia, trauma, heroin usage, and methanol intoxication [1].

Our patient presented with an uncommon diagnosis of bilateral basal ganglia infarction and comorbidities of STEMI, staying in the ICU. Cerebral vasospasm may have contributed to the infarction and caused vascular thrombosis via platelet aggregation, potentiated by cocaine [4]. Cocaine acts as a vasoconstrictor and alters the caliber of the vessels, which can cause severe restrictions of blood flow to the smaller vessels of the basal ganglia, as evidenced by the infarction of the basal ganglia that can be seen on diffusion-weighted MRI [5].

Cocaine induces secondary vasospasm that warrants imaging and toxicology reports for concurrent drug use. The patient tested positive for phenobarbital use, which could have exacerbated or encouraged vasospasm (Table 1) [6]. Further clinical manifestations expand to cardiac, pulmonary, psychiatric, neurologic, gastrointestinal, head and neck, endocrine, infections, weight loss, optic neuropathy, rhabdomyolysis, and arterial/venous thrombosis [6]. Phenobarbital use with cocaine use is commonly seen in patients who try to counteract the stimulant effect of cocaine to stay awake longer and ease stress [7]. There have been a few rare cases reported in the literature where bilateral cerebral ischemic injuries were found in patients with toxicology tests who tested positive for cocaine [1,5-7]. However, the literature does not present cases where further investigations were done for patients with cerebrovascular injuries in the setting of cocaine use, and the impact of medications used for intervention can further harm an individual due to interactions [5,6,8].

Research has encouraged viewing the lesions with newer imaging techniques, including PET and SPECT [8]. Studies have shown that intravenous cocaine administration lowers cerebral blood volume flow, which can be evident on PET and SPECT [9]. Cases of bilateral basal ganglia infarction due to cocaine have also been associated with symptoms of vasculitis in the small arteries of the deep white matter [10,11]. Thus, this encourages the need for toxicology studies in patients presenting with acute ischemic cerebral infarction, especially when images show such asymmetric lesions.

## Conclusions

Overall, our case presentation differs from other cases of bilateral basal ganglia infarction from cocaine use due to the comorbidity of STEMI, as well as HIV, hepatitis C, and acute kidney injury. The concurrent use of phenobarbital drugs is suspected to have encouraged further vasospasm as it may cause hypotension events. T2 FLAIR MRI brain imaging aided in this diagnosis of ischemic infarction in the basal ganglia. Further research is proposed for varying forms of imaging techniques, as well as emergent treatment measures.
